# Systems biology from virus to humans

**DOI:** 10.1186/s40543-015-0047-4

**Published:** 2015-02-05

**Authors:** Youri Lee, Yu-Jin Kim, Yu-Jin Jung, Ki-Hye Kim, Young-Man Kwon, Seung Il Kim, Sang-Moo Kang

**Affiliations:** Center for Inflammation, Immunity & Infection, Institute for Biomedical Sciences, Georgia State University, Atlanta, GA 30303 USA; Division of Life Science, Korea Basic Science Institute, Daejeon, 305-333 South Korea

## Abstract

Natural infection and then recovery are considered to be the most effective means for hosts to build protective immunity. Thus, mimicking natural infection of pathogens, many live attenuated vaccines such as influenza virus, and yellow fever vaccine 17D were developed and have been successfully used to induce protective immunity. However, humans fail to generate long-term protective immunity to some pathogens after natural infection such as influenza virus, respiratory syncytial virus (RSV), and human immunodeficiency virus (HIV) even if they survive initial infections. Many vaccines are suboptimal since much mortality is still occurring, which is exampled by influenza and tuberculosis. It is critically important to increase our understanding on protein components of pathogens and vaccines as well as cellular and host responses to infections and vaccinations. Here, we highlight recent advances in gene transcripts and protein analysis results in the systems biology to enhance our understanding of viral pathogens, vaccines, and host cell responses.

## Review

### Introduction

Viruses contain all the essential factors necessary for initiating infection and replication in a new target cell. Thus, information on the protein composition of a virus particle often serves as an initial guide in determining functional roles for viral proteins as well as antiviral and/or vaccine antigen target molecules. With advances in proteomics techniques and the availability of annotated genomic sequences for several mammalian species, the view that a virion is a minimal package of its genome and essential viral proteins for the first round of genome replication is being changed. Proteomic analysis of virions identified host proteins that are packaged into the virus particles along with the viral components (Table 1). In particular, enveloped viruses have the capability to incorporate numerous host proteins, both into the interior of the virus particles as well as into the lipid envelope (Cantin et al. 2005; Bortz et al. 2003; Johannsen et al. 2004; Kattenhorn et al. 2004; Zhu et al. 2005). Similarly, host proteins have been detected in vaccinia virions, human immunodeficiency virus (HIV) type 1, and Moloney murine leukemia virus (MoMLV) vector particles (Chung et al. 2006; Chertova et al. 2006; Saphire et al. 2006).

**Table 1:** **Proteomic analysis of representative host cell proteins incorporated into virus or virus-like particles**

**Virus**	Host proteins in virus or VLP*	
Influenza virus^a^	ADAR3, H2A, HSP90, nucleolin, ITGAV, hnRNPA1, glypican 4 (kglypican), ANXA4, CD9, CD81, cofilin 1, cyclophilin A, profilin, HSP27, integrin beta, ANXA11, CD59	
Respiratory syncytial virus^b^		
Cytoskeleton	B-actin, keratin, moesin, filamin-A, TUBA1B, TUBB, HSP70, HSP90-alpha,beta	
Energy pathways	ALPI, PK, GAPDH	
Immune response	Putative annexin A2-like protein, ANXA1, CD55	
H5N1 VLP^c^	ADP-ribosylation factor, alpha tubulin, Arp2/3 complex subunit, beta-tubulin 10, actin, 40S ribosomal protein S24, 60S acidic ribosomal protein P1,2, ribosomal protein L40, HSP90, heterotrimeric G-protein gamma subunit-like protein, putative Rho1, glutathione S-transferase sigma, ATPase subunit C, casein kinase II subunit alpha, fatty acid-binding protein, 14-3-3 zeta, Rbp1-like RNA-binding protein PB	
H5N1/H3N1 VLP^d^	b-tubulin, Myosin, ECM proteins integrin alpha, HSP90, HSP70, HSP27, annexin, tetraspanin, CD9, glycolytic enzymes enolase 1, pyruvate kinase, glyceraldehyde-3-phosphate dehydrogenase, unclassified proteins, aldo-keto reductase family 1, WD repeat domain 1, gamma-glutamyltransferase, peroxiredoxin 2	

^a^Influenza virus (H1N1,A/Cal/07/2009) was purified after growing in A549 cells (Shaw et al. 2008; Dove et al. 2012).

^b^Respiratory syncytial virus was purified after growing in A549 cells (Radhakrishnan et al. 2010).

^c^H5N1 VLPs (H5N1, A/Indonesia/102 5/2005) were purified after expressing in SF9 insect cells (Song et al. 2011).

^d^H3N2/H5N1 VLPs (H3N2, A/Taiwan/083/2006 and H5N1, A/Hanoi/30408/2005H5N1) were purified after expressing in Vero cells (Wu et al. 2010).

*Adenosine deaminase, RNA-specific, 3 (ADAR3), histone 2A (H2A), heterogeneous nuclear ribonucleoprotein A1 (hnRNP1), tubulin alpha-1B (TUBA1B), tubulin B (TUBB), intestinal type alkaline phosphatase (ALPI), heat shock protein 27 kDa (HSP27) peroxiredoxin (PRX), annexin A 4 (ANXA4), annexin A11 (ANXA11), annexin A11 (ANXA1), integrin alpha V (ITGAV), dynein 1, heat shock protein 90 kDa (HSP90), heat shock protein 70 kDa (HSP70), pyruvate kinase (PK), beta-actin (B-actin), alkaline phosphatase (ALPI), complement decay acceleration factor (CD55), glyceraldehyde-3-phosphate dehydrogenase (GAPDH).

It is expected that cellular proteins found within the virus particles would provide clues as to the virus assembly pathway and events that govern virus infectivity as well as vaccine development.

Vaccination is considered the most effective measure to prevent global infectious diseases. Smallpox and polio diseases are good successful cases of global threats that almost disappeared by effective global vaccination. However, there are still many infectious diseases that claim over 15 million deaths annually. Live attenuated influenza virus (LAIV) vaccine was approved in 2003 and is currently being used for human vaccination. LAIV is safe and effective in young children and adults (Rhorer et al. 2009). Recently, the use of noninfectious virus-like particles (VLPs) that self-assemble by spontaneous interactions of viral structural proteins has been suggested and developed as alternative approaches for developing advanced vaccines for a wide range of viruses that cause disease in humans (Roy and Noad 2009; Kang et al. 2009a; Kang et al. 2009b). It is worth noting that a VLP-based human papillomavirus (HPV) vaccine against HPV responsible for cervical cancer was produced in the yeast system and approved for the market in 2006 (Garland et al. 2007).

Influenza VLPs expressed by recombinant baculovirus (rBV) systems that present multi-component antigens, including HA and matrix 1 (M1), with or without NA, and that are capable of inducing cognate responses against homologous strains of influenza virus have been widely described (Roy and Noad 2009; Kang et al. 2009a; Kang et al. 2009b). In particular, 2009 H1N1 new pandemic, H5N1, and H7N9 avian influenza VLP vaccines were produced by the insect cell rBV expression system, and tested in clinical trials, demonstrating their safety and efficacy (Khurana et al. 2011; Lopez-Macias 2012; Lopez-Macias et al. 2011; Klausberger et al. 2014; Smith et al. 2013; Fries et al. 2013). Also, influenza VLPs were engineered to express highly conserved influenza virus M2 ectodomains and found to induce cross immunity to heterologous influenza virus strains (Kim et al. 2013a; Kim et al. 2014; Kim et al. 2013b).

Any cellular proteins that may be incorporated into viral particles are also likely to be present at very low levels. Mass spectrometry of tryptic peptides combined with database searching for identification is now becoming the preferred and advanced method for such proteomic studies revealing details on protein components even at a very low level (Table 1).

Vaccinology and immunology were born from the pioneering work of scientists such as Jenner and Pasteur over 200 years ago. Despite the common origins of vaccination and immunology concepts, the two disciplines have evolved in parallel directions independently. Immunologists remain largely ignorant about the mechanisms of how vaccines work. Meanwhile, vaccines were made empirically, and until recently, vaccinologists have shown little interest in the immunological mechanisms by which the vaccine confers protective immunity. Better understanding of immunological mechanisms of vaccination is expected to provide informative insights into the rationale design of future vaccines against difficult pathogens.

The innate immune system including dendritic cells (DCs), macrophages, and other immune cells senses viruses, bacteria, parasites, and fungi through pathogen-recognizing receptors (PRRs) (reviewed in (Coffman et al. 2010; Kawai and Akira 2007; Iwasaki and Medzhitov 2010)). The nature of the DC subtypes and the particular PRR triggered plays an essential role in modulating the strength, quality, and memory of adaptive immune responses (Pulendran and Ahmed 2006; Steinman 2008). More than 26,000 genes are estimated to be present in human genomes. Exposure to a pathogen or vaccination introduces changes in the expression of a substantial fraction of these genes. Systems biological tools offer an informative insight into the complex network of the immune system in our body. That is, high-throughput data on the genes, mRNAs, microRNAs, and proteins that constitute the biological networks are providing new information in our overall understanding of complex immune systems. Systems biology capitalizes on several ‘omic’ technologies that might define and monitor all the components of the systems. Thus, recent advances in the innate immune system and the use of systems biological approaches are providing powerful tools to reveal the basic mechanisms by which the innate immune system modulates protective immune responses to vaccination (Pulendran and Ahmed 2006; Steinman 2008).

This review focuses on proteomic components of some viruses and vaccines (Table 1), cellular responses of host target cells and immune cells upon exposure to virus and vaccines (Table 2), as well as *in vivo* gene expression profiles in animals and humans in response to respiratory viral infection or certain successful vaccinations.

**Table 2:** **Representative gene expression profiles in host cells as a result of virus infection or VLP stimulation**

**Host cells**	**Virus/VLPs**	Gene expression profiles in host cells*
MDCK^a^	Influenza	TUBA2, CK-8, B-actin, keratin 10, ANXA1, KCIP-1
VERO^b^	Influenza	Keratin1, 8, 10, tubulin, TUBA, RBBP4, ITGA3, HSP27, Ndr1, ANXA4, PK, GAPDH, HSP105, ITGAV, dynein 1, HSP90, ITGA3, HSP 70
A549^c^	RSV	HRNR, HIST2H2BE, HIST1H2BC, H2AFX, DTX3L, IFI35, SYNE1, SUMO2, MYADM, PARP14, VIM, C21ORF70, TFAM, POLDIP3, DEK, EFHD2, KIAA1967, ENO1, ENY2
BEAS-2B^d^ HBEC	RSV	ATP6V0D2, OTOA, SCNN1G, SMA5, CYP4F8, EPB41L4B, ELP2, PRKCE, HBP1, DUSP2, TUBB1, TRNT1, ELOVL5, PTPRG, MAP2K7, TSGA10, KRT12, SYN1, SIRPB1
MDDC^e^	HIV-VLP	Expression of genes involved in the morphological and functional changes characterizing the MDDCs activation and maturation: MEF2A, NFE2L2, DC-UbP, PGM3, DGKH, PTGS2, IL8, ARRB1, BTG3, SOSTM1, HLA-DOA - MHC class II, TADA3L, MTHFD2, PRKX, BAG3 KCNK10, member 10, PBEF1
PBMC^f^	HIV-VLP	Expression of genes involved in the morphological and functional changes characterizing the PBMCs activation and maturation: CTSL, IL3RA, SMOX, BCL2, G0S2, IER3, SERPINB2, LIMK2, IL6, IL8, PBFE1, PBX3, IL1-A,B, CCL3 (MIP 1α), CCL7, CCL18, CCL20, CXCL1, CXCL2, CXCL3, CXCL6, CXCL13, INHBA, ACTN1, AQP9, EMR1, SLC25A37, SLCO4A1, MAD, CCL4 (MIP1β)
RAW264.7^g^	HBc-VLP	HSP70, Eno1 protein chain A, peroxiredoxin 1, phosphoglycerate kinase, glutathione S-transferase A2, ferritin light chain 1, hypothetical protein, prohibitin, glyceraldehyde-3-phosphate dehydrogenase

^a^Madin-Darby canine kidney (MDCK) cells that were infected with influenza virus (H1N1, A/PR/8/34) (Vester et al. 2010).

^b^Vero (kidney epithelial cells) cells that were infected with influenza virus (H1N1, A/PR/8/34) (Vester et al. 2010).

^c^A549 (adenocarcinomic human alveolar basal epithelial cells) cells were infected with respiratory syncytial virus (Munday et al. 2010).

^d^BEAS-2B4 (human bronchial epithelial cell line, subclone S6), human bronchial epithelial cells (HBEC) were infected with respiratory syncytial virus (Huang et al. 2008).

^e^Human monocyte-derived dendritic cells (MDDCs) were stimulated with HIV-1 (clade A) Pr55gag virus-like particles (HIV-VLPs) (Arico et al. 2005).

^f^CD14+ human peripheral blood mononuclear cells (PBMCs) were stimulated with HIV-1 Pr55gag virus-like particles (HIV-VLPs) (Buonaguro et al. 2008).

^g^RAW264.7 (mouse macrophage cell line) cells were stimulated with hepatitis B virus core protein virus-like particles (HBc-VLPs) (Yang et al. 2008).

*Influenza: H1N1 (A/PR/8/34), HIV-VLp (HIV-1 Pr55gag virus), HBc-VLP (hepatitis B virus). Tubulin alpha (TUBA), protein kinase C inhibitor protein-1 (KCIP-1), nameretinoblastoma binding protein 4 (RBB4), serine/threonine kinase 38 (Ndr), MADS box transcription enhancer factor 2 (MEF2A), nuclear factor-like 2 (NFE2L2), dendritic cell-derived ubiquitin-like protein (DC-UbP), phosphoglucomutase 3 (PGM3), diacylglycerol kinase (DGKH), prostaglandin-endoperoxidase synthase 2 (PTGS2), interleukin 8 (IL-8), arrestin beta 1 (ARRB1), BTG familymember 3 (BTG3), sequestosome 1 (SOSTM1), MHC class II (HLA-DOA), butyrate-induced transcript (HSPC121), transcriptional adaptor 3 (TADA3L), Mst3 and SOK1-related kinase (MST4), UDP-Gal:betaGlcNAc bea 1.4-galactosyltransferase (B4GALT5), NAD-dependent methylenetetrahydrofolate dehydrogenase (MTHFD2), small glutamine-rich tetratricopeptide repeat (SGTB), protein kinase (PRKX), Nedd4 binding protein 1(X-linked N4BP1), pellino homologue 1(PELI1), sterol-C4-methyl oxidase-like (SC4MOL), BCL2-associated athanogene 3 (BAG3), potassium channel, subfamily K (KCNK10), pre-B-cell colony enhancing factor 1 (PBEF1), hornerin (HRNR), H2B type 2-E (HIST2H2BE), H2B type 1-C/E/F/G/ (HIST1H2BC), H2A (H2AFX), protein deltex-3-like (DTX3L), interferon-induced 35 kDa protein (IFI35), Nesprin-1 (SYNE1), small ubiquitin-related modifier 2 (SUMO2), myeloid-associated differentiation marker (MYADM), poly [ADP-ribose] polymerase 14 (PARP14), vimentin (VIM), uncharacterized protein C21orf70 (C21ORF70), transcription factor A, mitochondrial (TFAM), polymerase d-interacting protein 3 (POLDIP3), protein DEK (DEK), EF-hand domain-containing protein D2 (EFHD2), protein KIAA1967 (KIAA1967), a-enolase (ENO1), enhancer of yellow 2 transcriptionfactor homologue (ENY2), ATPase, H+ transporting, lysosomal 38 kDa, V0 subunit d isoform 2 (ATP6V0D2), otoancorin (OTOA), sodium channel non-voltage-gated 1 gamma (SCNN1G), cytochrome P450 family 4 subfamily F polypeptide 8 (CYP4F8), erythrocyte membrane protein band 4.1 like 4B (EPB41L4B), signal transducer and activator of transcription 3 interacting protein 1(ELP2), protein kinase C epsilon (PRKCE), HMG-box transcription factor 1 downregulated genes (HBP1), dual specificity phosphatase 2 (DUSP2), tubulin beta 1 (TUBB1), tRNA nucleotidyl transferase CCA-adding 1 (TRNT1), ELOVL family member 5 elongation of long chain fatty acids (ELOVL5), protein tyrosine phosphatase receptor type G (PTPRG), mitogen-activated protein kinase 7 (MAP2K7), testis specific 10 (TSGA10), keratin 12 ( KRT12), synapsin I (SYN1), signal-regulatory protein beta 1 (SIRPB1), glucuronidase, beta pseudogene (SMA5), EGF-like module containing, mucin-like, hormone receptor-like sequence 1(EMR1).

### Cellular and viral proteins within virus and virus-like particles

#### Influenza virus

With advances in proteomic analysis and studies, a virion is not simply a package of its genome and viral proteins. Numerous host cellular proteins were found to be incorporated into virus particles in particular enveloped virions. HIV virions were found to incorporate Tsg101, cyclophilin A, and APOBEC3G in addition to their viral proteins (Chertova et al. 2006; Saphire et al. 2006; Demirov et al. 2002; Franke et al. 1994; Mariani et al. 2003). Tsg101 host protein was reported to play a crucial role in virus assembly of HIV whereas cyclophilin A modulates HIV-1 infectivity and APOBEC3G is an anti-viral factor that promotes hypermutation of the viral genome (Franke et al. 1994; Mangeat et al. 2003). Among many other cellular proteins, identification of these three host proteins has increased our understanding of how HIV would interact with its host proteins. Nine viral proteins out of the 11 influenza A virus proteins are present in the influenza virion (PB1, PB2, PA, HA, NP, NA, M1, M2, NEP). The glycoproteins hemagglutinin (HA) and neuraminidase (NA) are embedded into the lipid envelope of the influenza virus particle and form the spikes visible under the electron microscope. The ion channel protein M2 is also found within the virion but at a lower level. The matrix M1 protein lies beneath the viral membrane, surrounding the ribonucleoproteins, which consist of eight viral RNA segments coated with the nucleoprotein (NP) and bound by the trimeric polymerase complex (PB1, PB2, PA). The nuclear export protein (NEP) is also found within influenza virus particles.

By utilizing mass spectrometry of tryptic peptides (multidimensional protein identification technology liquid chromatography-tandem mass spectrometry (LC-MS/MS) analysis) combined with database searching for identification, 36 host-encoded proteins were identified in the influenza A/WSN/33 (H1N1) virus particles that were grown in Vero (African green monkey kidney) cells (Shaw et al. 2008). The following host cell proteins are also found in other enveloped viruses. (i) Cytoskeletal proteins: Cytoskeletal proteins such as tubulin and actin were present in the interior of influenza virions which most likely reflects their active participation in moving the viral components to the assembly site and possible cytoskeletal reorganization that occurs during bud formation. (ii) Annexins: Several annexin family members (A1, A2, A4, A5, and A11) were found in influenza virus particles. Annexins are calcium-dependent phospholipid-binding proteins and are proposed to act as scaffolding proteins at certain membrane domains. (iii) Tetraspanins: Two members of the tetraspanin family, CD9 and CD81, were present within influenza virions and are most likely inserted into the viral envelope. Tetraspanins have four transmembrane domains and two extracellular loops and are involved in specialized membrane domains. Tetraspanins (CD9 and CD81) have also been implicated in both fusion and egress pathways for a number of other viruses (Ho et al. 2006; Jolly and Sattentau 2007). (iv) Cyclophilin A: Cyclophilin A (a peptidyl-prolyl isomerase) was shown to be in the core of the influenza virion and in other different viruses. There is a precedent for the involvement of cyclophilin proteins in replication of different viruses. (v) CD59: CD59 is a complement regulatory protein that acts by inhibiting formation of the membrane attack complex. CD59 (glycosylphosphatidylinositol membrane anchored protein) was found to be associated the influenza virus envelope. Enveloped viruses are susceptible to direct complement-mediated lysis and incorporating CD59 (with DAF and CD46) into lipid envelope is considered to play a protective role from membrane attack by the host complement system. This has important implications for virus host-range. The virus produced and transmitted within the same host species would be protected since complement control proteins are highly species specific. However, virus transmitted to another host species would become susceptible to lysis by the complement system from a different host. (vi) Metabolic enzymes: Proteomic analysis of influenza virus particles identified a number of proteins involved in the glycolytic pathway (pyruvate kinase, enolase 1, GAPDH glyceraldehyde-3-phosphate dehydrogenase, phosphoglycerate kinase). It is possible that some of these cellular proteins might have other additional functions such as viral RNA genome transcription.

#### Respiratory syncytial virus

Respiratory syncytial virus (RSV) is the most important respiratory virus causing lower respiratory tract infection in young children and neonates. There is no vaccine against RSV. In contrast to influenza virus, formalin inactivated RSV (FI-RSV) in alum adjuvant formulation is known to cause vaccine-enhanced respiratory disease (Kapikian et al. 1969; Kim et al. 1969). Reinfections are common throughout life, indicating that natural RSV infection fails to establish long-lasting immunity (Hall et al. 1991; Piedra 2003; Bont et al. 2002; Nokes et al. 2008; Scott et al. 2006; Glezen et al. 1986). Identification of protein components of RSV might provide a unique clue to therapeutic intervention or vaccine design targets.

The RSV A2 strain was produced in the human respiratory airway cell-line Hep2 and purified using sucrose gradient ultracentrifugation. The RSV protein components were analyzed by one-dimensional nano-LC-MS/MS, resulting in identification of 26 cellular proteins in addition to all the major virus structural proteins (Radhakrishnan et al. 2010). Representative host cell proteins associated with purified RSV particles include proteins associated with the cortical actin network, energy pathways, and heat shock proteins (HSP70, HSC70, and HSP90). In particular, the HSP90 protein was suggested to play an important role in the RSV assembly process. The presence of virus-associated actin network proteins as well as cofilin-1, caveolin-1, and filamin-1 in the mature virus may indicate an important role in RSV assembly in lipid raft microdomains and in maintaining the RSV architecture. Unlike other viruses, high levels of heat-shock proteins associated with RSV particles remain unclear for their significance.

#### Protein components of VLPs as vaccine candidates

Influenza VLP vaccines that were produced using the rBV-insect cell expression system were demonstrated to be safe and immunogenic in the clinical trials of healthy adults (Khurana et al. 2011; Lopez-Macias et al. 2011). Insect cell culture derived influenza VLP vaccines were shown to be more immunogenic compared to the conventional egg-substrate split vaccines (a Phase II human clinical trial of the trivalent seasonal influenza VLP vaccine candidate, Novavax, Inc.). These clinical studies demonstrated the safety and efficacy of VLP vaccines produced in insect cells using the rBV expression system. Thus, it is highly significant to have information on protein components of VLP vaccines. Using one-DE-LC-MS/MS technology, comprehensive proteomic analysis of the insect cell derived, rBV expressed influenza H5 VLPs identified viral proteins as vaccine target antigens as well as 37 additional host-derived proteins (Song et al. 2011). Many of host-cell-derived proteins in influenza VLPs are known to be present in other enveloped viruses and involved in different cellular structures and functions including those from the cytoskeleton, translation, chaperone, and metabolism. Influenza H5 VLPs produced in insect cells were found to be associated with host proteins involved in actin cytoskeleton, vesicular trafficking (ADP-ribosylation factor, vesicle-associated membrane proteins, vacuolar protein sorting 28, myosin II essential light chain), heat-shock protein 90, ribosomal proteins, putative ubiquitin/ ribosomal protein S27Ae fusion protein, and cell-signaling-related proteins (heterotrimeric guanine nucleotide binding protein gamma subunit-like protein, Rho1). As expected, many rBV vector-derived proteins were also found to be in H5 VLPs. These structural proteins originated from rBV include occlusion derived and polyhedron associated proteins (AcOrf-102, −114), capsid or capsid associated proteins, and baculovirus envelope proteins.

Mammalian influenza VLPs may more closely mimic authentic virions in their morphology, in functional HA, and in other molecular constituents. Stably transformed Vero cells expressing influenza M1, M2, HA, and NA were used to produce mammalian influenza H3N2 VLPs and H5N1 VLPs (Song et al. 2011). Proteomic analysis of mammalian VLPs using LC-MS/MS technologies identified 22 VLP-associated cellular proteins that are analogous to those cellular proteins commonly found in the influenza virions (Song et al. 2011). These cellular proteins incorporated into mammalian influenza VLPs include cytoskeleton proteins, extracellular matrix proteins, heat shock proteins, annexins, tetraspanins, clathrin heavy chain, and glycolytic enzymes. Thus, the cellular proteins identified in VLPs without viral genomes are important in the normal virus life cycle during virus assembly and budding from the host cells.

### Host cell gene expression profiles upon viral infections or in response to VLPs

A proteomic approach applying the quantitative 2-D DIGE and nanoHPLC-nanoESI-MS/MS analysis was used to investigate the dynamic cellular host cell response induced by influenza virus infection in two different cell lines, Madin-Darby canine kidney (MDCK) and Vero cells. Upon influenza virus (A/PR/8/34) infection, changes in gene expression of MDCK infected cells were observed in the interferon (IFN)-induced signal transduction, cytoskeleton remodeling, vesicle transport, and proteolysis (Vester et al. 2010). In Vero cells infected with influenza virus, alterations of gene expression include heat shock and oxidative stress response-related proteins.

To gain an understanding of the RSV associated host cell gene expression, differentially expressed genes in human respiratory epithelial cells (A549) were determined by cDNA microarray analysis after RSV infection (Martinez et al. 2007). Among 85 genes that were up-regulated at early times post infection (0 to 6 h post infection (pi)), most highly expressed genes are involved in chemotaxis, inflammation, and some integrins. Genes related to IFN-stimulation and NF-ƙB pathways were up-regulated between 6 and 12 h pi. At later times post infection, immune response-related genes were expressed at high levels. These findings suggest a temporal relationship between RSV infection and the host response to RSV replication. Supplementary validation experiments using conventional methods are required to confirm these findings.

HIV-1 gag virus-like particles (HIV-VLPs) produced by the recombinant baculovirus expression system was used to stimulate CD14^+^ monocyte-derived dendritic cells (MDDCs) enriched from peripheral blood mononuclear cells (PBMCs) of normal healthy donors (Buonaguro et al. 2008). Genomic transcriptional profile of HIV-VLPs activated MDDCs revealed high expression of genes that are responsible for activation and maturation of MDDCs. Representative genes up-regulated include antigen processing and presentation (IL3RA, BCL2), cell shape and extracellular matrix, chemokine and cytokines (IL-6), cytokine network (IL1A, B), cytokine-receptor interactions, immune response membrane proteins, chemokine receptors (CCL3, 4), and Toll-like receptor (TLR) signaling pathway (IL8). Similar data of gene expression profile were also reported to be observed using PBMCs activated by HIV-VLPs (Buonaguro et al. 2008). The same group of study demonstrated that the effects of HIV-VLPs on MDDCs are not mediated through TLR2 and TLR4 signaling (Buonaguro et al. 2006). Also, influenza VLP-loaded MDDCs that were obtained from human healthy donors were demonstrated to be effective in generating functional CD8 T cells (Song et al. 2010), implicating that VLP vaccines can induce both humoral and cellular host immune responses.

Hepatitis B virus core antigen VLP (HBc-VLP)-pulsed and control macrophage cells (RAW264.7) were subjected to two-dimensional electrophoresis and tandem MS (Yang et al. 2008). Analysis of differentially expressed proteins revealed that heat-shock protein 70 and prohibitin in addition to proteins in the glycolytic pathway were highly up-regulated upon stimulation with HBc-VLPs. It is speculated that stress-response proteins (HSP70, prohibitin) may contribute to the uptake, processing, and presentation of VLP vaccine particles.

### Animal models and systems biology to better understand pathogenesis and vaccination

The dynamics of virus pathogenesis are multifaceted and can be better comprehended by looking at the system as a whole. Human patients with highly pathogenic avian influenza H5N1 virus typically develop a viral primary pneumonia progressing rapidly to acute respiratory distress syndrome (Abdel-Ghafar et al. 2008). An aberrant immune response is thought to play a significant role in the severe respiratory disease that may ultimately lead to death (Peiris et al. 2009). The term ‘cytokine storm’ referring to an uncontrolled inflammatory response is often associated with H5N1 virus pathogenesis (Tisoncik et al. 2012). Human patients infected with H5N1 virus were shown to have high serum levels of pro- and anti-inflammatory cytokines (IL-6, IL-10, IFN-γ), macrophage and neutrophil chemoattractant chemokines (CxCL10, CXCL2, IL-8) (de Jong et al. 2006; Peiris et al. 2004; To et al. 2001). Host response to influenza H5N1 virus has been investigated in non-human primate, mouse, and ferret models. Global transcriptional profiling of infected lungs has revealed that virulence of influenza virus is associated with increased early and sustained inflammatory responses. Genes that showed correlates with disease severity during H5N1 virus infection include inflammasome components, viral sensing, neutrophil activation, NF-κB signaling, and chemokine signaling (Ibricevic et al. 2006; Cilloniz et al. 2010; Baskin et al. 2009; Cameron et al. 2008; Chang et al. 2011; Shinya et al. 2012).

It is suggested that lung homeostasis is lost when the innate immune system reached a high level of activation but was unable to contain the pathogen before viral cytopathicity (Boon et al. 2011; Sanders et al. 2011). The depletion of innate immune cell types lowered inflammatory cytokine levels in mouse lung homogenates but resulted in elevated lung viral titers, systemic virus spread, and reduced survival (Tumpey et al. 2005). Mice with decreased myeloid infiltrates and lack of NLRP3 inflammasome activation exhibited high susceptibility to influenza infection (Allen et al. 2009; Thomas et al. 2009). Selective neutrophil targeting in infected mice caused enhanced mortality (Tate et al. 2009). Therefore, innate inflammatory cells have host-beneficial functions rather than a primary causal role in pathology (Brincks et al. 2008; Tate et al. 2012).

An alternative view is that lung function is largely dysregulated through the damaging effects of leukocytes on epithelial and endothelial cells (Aldridge et al. 2009; Le Goffic et al. 2006; Lin et al. 2008). In support for this idea, monocyte-derived inflammatory macrophages and dendritic cells contributed to fatality (Lin et al. 2008). The relative pathogenic contributions of direct viral cytopathic damage versus dysregulated host inflammatory responses to lethal influenza infections remain as an important question to be answered. By using extensive microarray analysis of multigene transcriptional signatures from infected mouse lungs, a recent study suggested that differential activation of inflammatory signaling networks distinguished lethal from sublethal infections. From combined flow cytometry and gene expression analysis of isolated cell subpopulations from infected mouse lungs showed that neutrophil influx was largely responsible for the predictive transcriptional signatures. Together with these gene expression and flow data, automated imaging analysis identified chemokine-driven proinflammatory neutrophils, which might be activated by lethal viral loads. In line with these data, attenuation, but not ablation, of the neutrophil-driven response was shown to improve survival without changing viral spread. These findings with a possible a roadmap for the systematic dissection of infection-associated tissues support evidence for the primary contribution of damaging innate inflammation to some forms of influenza-induced lethality and provide. To more clearly differentiate host protective from damaging immunity, comprehensive data sets at both the organ and the cell level are needed.

As described above, RSV is also an important human respiratory pathogen against which there is no vaccine. Systemic gene expression signatures have been examined in lungs of mice infected with RSV (Pennings et al. 2011; Janssen et al. 2007; Tripp et al. 2013). A robust transcriptional response of interferon-associated and innate immunity genes was observed at day 1 (pi) but was reduced by day 3 pi, and the peak lung transcriptional response preceded the peak of viral replication. Host genes that were expressed were diverse and involved in the IFN response, inflammation, chemoattraction, and antigen processing. In particular, cytokine genes such as IL-1β orchestrate the proinflammatory response while others including intracellular reactive oxygen species (ROS) were effectors of inflammation (Janssen et al. 2007; Tripp et al. 2013; Segovia et al. 2012).

Host innate immune responses in response to vaccination are relatively not well understood yet. The live attenuated yellow fever vaccine 17D (YF-17D) is considered one of most effective vaccine with a 65-year history. YF-17D was shown to activate human monocyte-derived DCs by up-regulating CD80, CD86 markers and inflammatory cytokines (IL-6, TNF-α), and chemokines (IP-10, MCP-1) (Querec et al. 2006). Using CD11c^+^ DCs derived from mutant mice, YF-17D was found to stimulate DCs via multiple TLRs 2, 7, 8, and 9 to elicit the proinflammatory cytokines IL-12p40, IL-6, and IFN-α. Mice with a transfer of OT-I T cell receptor transgenic T cells and inoculated with YF-Ova8 induced a mixed T helper cell (Th)1/Th2 cytokine profile and CD8 T cells. Thus, effective vaccines may need to activate multiple innate immune components.

### Systems biology in humans for better understanding of disease and vaccination

#### Systems biology in humans with respiratory viral infections

Systems biology is a newly advancing field that uses an interdisciplinary approach aimed at understanding and predicting the properties of a living system through systematic quantification of all its components and intensive mathematical and computational modeling. Each component of the system is measured using high-throughput ‘omic’ techniques and in theory examined from the cellular level to the whole organism. Systems technologies include transcriptomic (microarray gene expression), modern mass spectrometry (proteomics, lipidomics, metabolomics), genomics, and protein-DNA interaction (chromatin immunoprecipitation).

A recent study (Mejias et al. 2013) reported whole blood gene expression profiles of microarray data to assess disease severity in infants (ages <6 months, 2 to 24 months) with respiratory syncytial virus infection in comparison with influenza and human rhinovirus (HRV), attempting to identify biomarkers that can objectively predict RSV disease severity. Despite the fact that influenza, RSV, and HRV infect common respiratory tracts, this study demonstrated that the degree of activation/suppression of specific immune-related genes was markedly different. Influenza stimulated a stronger activation of interferon, inflammation, monocyte, and innate immune response genes compared with RSV and rhinovirus. Neutrophil-related genes were significantly overexpressed in patients with RSV, followed by patients with rhinovirus, and were at a lower level in patients with influenza. Interestingly, RSV was associated with marked suppression of genes involved in B cell, T cell, lymphoid lineage, and antimicrobial responses. In contrast, this suppression was significantly milder or absent in children with influenza and rhinovirus.

The overexpression of interferon and innate immunity genes was similar in children with moderate and severe RSV but greater than that in children with mild RSV disease. The overexpression of neutrophil, monocyte, and innate immunity genes induced during the RSV acute disease faded over time. However, T cell lymphoid lineage and antimicrobial response genes were suppressed during the acute phase and then recovered back to normal levels. Remarkably, the suppression of B cell genes was persistent when patients' samples were analyzed 1 month after the acute infection. This low level of B cell genes might explain partially the less protective antibody responses after acute RSV infection. This study also indicates that RSV suppressed both the adaptive and innate responses more severely in younger infants less than 6 months old. Children with severe RSV demonstrated significantly greater underexpression of genes associated with T cells, cytotoxic and NK cells, and plasma cells.

Influenza triggers a more robust immune response than RSV, with greater induction of respiratory and systemic cytokines (Garofalo et al. 2005; Gill et al. 2008; Welliver et al. 2007). Antiviral responses against influenza and RSV were shown to be correlated with the interferon signature gene expression from peripheral blood mononuclear cells isolated from patients with acute influenza or RSV bronchiolitis (Ioannidis et al. 2012). This study provides evidence of the profound systemic dysregulation of both the innate and adaptive immune response induced by RSV infection in children and supports systems biology of gene expression profiling as a practical and powerful strategy to objectively stratify children with viral infection such as RSV. Nonetheless, these observations will require further analysis, as they may have implications for RSV vaccine development.

#### Systems vaccinology

One potential application of systems biology is to predict vaccine efficacy in humans. Molecular patterns or signatures of genes in the blood after vaccination might predict the later development of protective immune responses, representing a strategy to prospectively determine vaccine efficacy. Blood cells provide a snap-shot of many lineages and differentiation states within the immune system including the sites of vaccination. Microarray analyses using the Affymetrix Human Genome U133 Plus 2.0 array of total PBMCs revealed a molecular signature comprised of genes that are involved in innate sensing of viruses and antiviral immunity, in most of the vaccines.

YF-17D is a live-attenuated yellow fever virus, one of most effective successful human vaccines ever developed (Querec et al. 2009; Gaucher et al. 2008) and would provide an excellent model for systems vaccinology study in humans. YF-17D single vaccination induces antigen-specific CD8^+^ T cells and neutralizing antibody responses in humans that persist for several decades (Pulendran 2009; Pulendran et al. 2013). Recent studies (Querec et al. 2009; Gaucher et al. 2008) reported transcriptomic analysis of PBMCs isolated 3 to 7 day-post-vaccination of healthy adults with YF-17D. In these studies, a pattern of gene expression profile was revealed, which consists of genes encoding proteins involved in antiviral sensing and viral immunity, including the type I IFN pathway. It seems to be that the YF-17D vaccine is mimicking an acute viral infection.

Using computational analysis, signatures of gene expression in human PBMCs after vaccination appeared to be correlated with the magnitude of the antigen-specific CD8^+^ T cell and neutralizing antibody responses afterward (Querec et al. 2009). The functional relevance of one of the genes within the predictive signatures was speculated from machine-learning techniques to validate the predictive capacity (ref 22). Eukaryotic initiation factor-α kinase 4 (*EIF2AK4*) would be involved in programming professional antigen presenting cells (DCs) to stimulate CD8^+^ T cell responses (Querec et al. 2009). A *TNFRSF17* gene signature was predicted to be correlated with neutralizing antibody responses. *TNFRSF17* encodes the receptor for the B-cell growth factor BLyS-BAFF known to play a key role in the differentiation of plasma cells (Vincent et al. 2013).

The potential application of systems vaccinology in humans was further extended by studies on immunity to human influenza vaccines, the trivalent inactivated seasonal influenza vaccine (TIV) (Nakaya et al. 2011; Bucasas et al. 2011; Franco et al. 2013), and LAIV (FluMist) (Nakaya et al. 2011). TIV is the most common flu shot vaccine, which is a mixture of inactivated split H1N1, H3N2, and influenza B vaccines. To determine whether molecular signatures after YF-17D vaccination would be similar to other vaccines such as influenza, a recent study carried out a systems analysis of responses to TIV and LAIV in young healthy adults during three consecutive influenza seasons (Nakaya et al. 2011). The group of people who received TIV showed higher antibody titers and more plasmablasts compared to the group who received nasal spray of LAIV. As expected from the fact that replicating LAIV infects mucosal tissues of respiratory tracts, humans with a nasal spray of LAIV showed a robust type I IFN antiviral transcriptomic signatures. TIV-vaccinated humans also expressed some gene encoding type I interferons and related proteins as well as gene encoding proinflammatory mediators (Nakaya et al. 2011). In this study, genes that are involved in innate sensing of viruses and antiviral responses were highly expressed within 1 to 3 days after vaccination of humans (Nakaya et al. 2011). After 3 to 7 days of vaccination, the up-regulated genes (*TNRSF17*, *XBP-1*) were found to be involved in the differentiation of plasmablasts, which is likely to be correlated with the magnitude of the later hemagglutin titers (Nakaya et al. 2011). Other studies also demonstrated a plausible correlation between this ‘plasmablast signature’ and its capacity to predict antibody titers (Obermoser et al. 2013; Furman et al. 2013; Tsang et al. 2014).

## Conclusions

In summary, recent advances in applying systems-level approaches to virus, vaccines, cells, organs, animals, and even to humans revealed extensive new information on gene expression and protein components, thus showing some promises in future. This new information extend our understanding in the pathogen-host cell interactions, host cellular responses, disease pathogenesis, host immune responses, and eventually new therapeutics and novel vaccine development. However, it should be reminded that microarray data may fail to provide convincing significance in our complex biological systems. In addition, the results of systems analysis need to be validated by experiments generating functional data such as protein techniques, gene perturbation, or deficient animal models. Finally, systems biology requires multidisciplinary and close collaborative experts including biologists, vaccinologists, immunologists, systems bioinformatics, computational specialists, and clinicians.
